# Targeting host-specific metabolic pathways—opportunities and challenges for anti-infective therapy

**DOI:** 10.3389/fmolb.2024.1338567

**Published:** 2024-02-22

**Authors:** Monika I. Konaklieva, Balbina J. Plotkin

**Affiliations:** ^1^ Department of Chemistry, American University, Washington, DC, United States; ^2^ Department of Microbiology and Immunology, Midwestern University, Downers Grove, IL, United States

**Keywords:** lipid enzymes, LCAT, LPL, host-directed therapy, intracellular pathogens, anti-infectives, drug development

## Abstract

Microorganisms can takeover critical metabolic pathways in host cells to fuel their replication. This interaction provides an opportunity to target host metabolic pathways, in addition to the pathogen-specific ones, in the development of antimicrobials. Host-directed therapy (HDT) is an emerging strategy of anti-infective therapy, which targets host cell metabolism utilized by facultative and obligate intracellular pathogens for entry, replication, egress or persistence of infected host cells. This review provides an overview of the host lipid metabolism and links it to the challenges in the development of HDTs for viral and bacterial infections, where pathogens are using important for the host lipid enzymes, or producing their own analogous of lecithin-cholesterol acyltransferase (LCAT) and lipoprotein lipase (LPL) thus interfering with the human host’s lipid metabolism.

## Introduction

Lipids are essential for a wide range of cell maintenance activities including, energy production and storage, vesicle transport and participation in immune signaling (Horn and Jaiswal, 2019; Jarc and Petan, 2019; Herker et al., 2021). Intracellular pathogens have highjacked the use of host lipids throughout infection by adapting processes for utilization of a number of host lipids, thereby supporting the pathogen’s replication and sustainability (Allen and Martinez, 2020). The replication of these intracellular microbes, both facultative and obligate, progresses via their regulation: 1) of host cell exogenous lipid uptake, resulting in pathogen-altered host lipid composition; and, 2) expression of host enzymes involved in lipid biosynthesis (Toledo and Benach, 2015; Gago, Diacovich, and Gramajo, 2018; Lin et al., 2020; O’Neal et al., 2020; Theken et al., 2021). Advantages to high jacking and altering host lipid composition by pathogens is evolutionarily beneficial to the pathogen since it takes over multiple biosynthetic and physiologic processes that could be metabolically costly. For example, these can include fundamental functions such as energy production through the utilization of stored and/or free lipids including lipid droplets (LDs), and free fatty acids. ATP from these substrates is produced by various processes such as, phospholipase selective catabolism, lipolysis, and lipophagy (Saito et al., 2019). Pathogenic microbes also exhibit the capability to utilize these catabolic pathways through application of their prokaryotic phospholipases, which also allows them to generate energy by intracellularly using a combination of the release and sequestration of metabolites, thus resulting in the generating of their own ATP. Furthermore, shifts in energy production through regulation of host lipid metabolism is also implicated in affecting host cell persistence, pathogen longevity, and downregulation of inflammation. This pathogen regulation of host lipid metabolism appears to play a role in their persistence within host cells, and downregulation of inflammation in addition to energy production.

Along with energy production, intracellular pathogens can utilize highly organized membrane regions, i.e., lipid rafts, for entry into the host cell. Lipid rafts within host membranes function as critical organizational areas that play an important role in signaling pathway regulation by enabling receptors to associate with external and internal stimuli. In addition to their cell-cell communication function, lipid rafts are also responsible for the release of fatty acid molecules, e.g., arachidonic and polyunsaturated fatty acids (PUFA), as lipid droplets (Toledo and Benach, 2015; Danielli et al., 2023). These acids serve as precursors for important host immune modulators including prostaglandins and leukotrienes, which in turn effect symptomatology during the infectious process (Noverr et al., 2003; Roingeard and Melo, 2017; Ripon et al., 2021). In addition to host lipid rafts, synthesis of intracellular pathogen membranes can have distinctive features with respect to lipid composition, with some intracellular pathogens expressing unique lipids within their membranes, unlike common bacterial lipids, as well as their role in host signaling pathways. With regards to some pathogen-associated cell membranes, it is interesting that these lipids are unable to be synthesized by the pathogen’s biosynthetic enzymatic systems, making it likely that these lipids are host-acquired (Toledo and Benach, 2015; Lin et al., 2020).

The three primary blood lipids are triacylglycerols, phospholipids, and cholesterol. Of these, triacylglycerols and cholesterol are the focus of pharmaceutical cardiovascular drug development. Triacylglycerols (TGs) are a high-energy source that account for more than three-quarters of the fuel needed for heart and liver function, which is accomplished by oxidation of TG’s long fatty acyl chains (Cox and Nelson, 2005)**.** With regards to cholesterol, it plays an essential role in mammalian cytoplasmic membrane integrity together with phospholipids, as well as bile acid and steroid synthesis. Because of the potential for off-label usage of the large and growing number of drugs targeting the initiating and potentiating factors associated with cardiovascular disease, the focus of this review is on key enzymes involved in cholesterol and tryglycerides metabolism, respectively, specifically lecithin-cholesterol acyltransferase (LCAT)- and lipoprotein lipase (LPL)-activating compounds.

The enzyme LCAT links phospholipid acyl chains to cholesterol molecules, thus, produces cholesterol esters (CEs). These esters, which are predominantly formed in high-density lipoprotein (HDL) particles, are essential in HDL-mediated reverse cholesterol transport (RCT) wherein intracellular cholesterol with phospholipids are transported to the liver (Feingold, 2022). In addition, HDL particles also remove cholesterol from lipid-loaded macrophages which play an essential role in initiation of atherosclerotic plaques (Lewis and Rader, 2005). Thus, the RCT efficacy is believed to be associated with coronary heart disease development (Haase et al., 2012; Peloso et al., 2014; Rader and Hovingh, 2014). In addition to RCT effectiveness, lipid homeostasis plays an important role in cardiovascular disease (CVD). The enzyme that is a major player in lipid homeostasis by mediating lipolysis of triacylglycerol rich lipoproteins is LPL. Dyslipidemia can occur when LPL malfunctions leading to hypertriacylglycerolemia (hypertriglyceridemia, chylomicronemia), an independent risk factor for CVD due to plasma accumulation of chylomicrons and very low-density lipoproteins (VLDL).

The focus of this review is to address the link between the infectious processes of pathogenic bacterial, parasites, and viruses, and the utilization, with or without alteration, of LCAT and LPL during human lipid homeostasis and dysmetabolism. This review first addresses current knowledge on the alteration and use of LCAT and LPL during human lipid homeostasis and dysmetabolism. It then addresses the links between the enzyme’s respective pathways and microbial infections as well as the relationship between the presence of the eukaryotic or eukaryotic-like LCAT/LPL in bacteria and parasites, and the opportunities and challenges associated with antimicrobial drug development.

## Roles of eukaryotic LCAT and LPL in host lipid metabolism—a brief overview

Fatty acids and monoacylglycerols that are absorbed through the intestinal cells postprandially are utilized to synthesize triacylglycerols. The key enzymes required for triacylglycerol synthesis are monoacylglycerol acyltransferase (MGAT) and diacylglycerol transferase (DGAT). MGAT catalyzes the addition of a fatty acid to monoacylglycerol, while DGAT catalyzes the addition of a fatty acid to diacylglycerol resulting in triacylglycerol formation. The majority of the cholesterol absorbed by the intestine is esterified to cholesterol esters by acyl-CoA cholesterol acyl transferase (ACAT). The triacylglycerols and cholesterol esters are subsequently packaged into chylomicrons in the endoplasmic reticulum of the intestinal cells. The size and composition of the chylomicrons formed in the intestine are dependent on the amount of fat ingested and absorbed by the intestine and the type of fat absorbed. Increased fat absorption results in larger chylomicrons (Wolska and Remaley, 2021; Feingold, 2022).

### LPL metabolizes the chylomicrons

**FIGURE 1:**
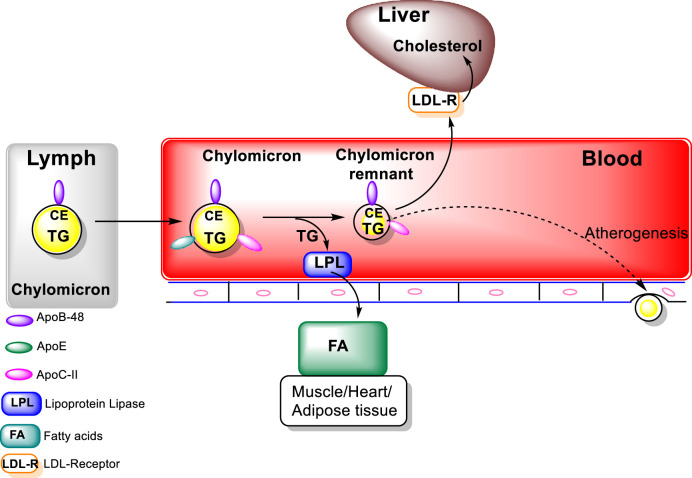
Exogenous Lipoprotein Pathway (figure modified from “Introduction to Lipids and lipoproteins”. K.R.Feingold, https://www.ncbi.nlm.nih.gov/books/NBK305896/).

Chylomicrons which are triacylglycerols rich particles are secreted into the lymph and delivered via the thoracic duct to the circulation, which facilitates delivery of the nutrients contained in chylomicrons to muscle and adipose tissue (Figure 1). LPL itself is synthesized at high levels in muscle and adipocytes then transported to the luminal surface of capillaries. Lipase maturation factor 1 (LMF1) plays a key role in the stabilization and movement of LPL from muscle cells and adipocytes to the capillary endothelial cell surface. (LMF1) is a protein bound to the endoplasmic reticulum (ER) membrane which functions in the maturation, i.e., folding and assembly LPL, among others (hepatic lipase (HL) and endothelial lipase (EL). High-density lipoprotein binding protein 1, anchored by glycosylphosphatidylinisitol (GPIHBP1), binds LPL, transports it to the capillary lumen then, anchors LPL to the capillary endothelium. Activation of LPL by Apo C-II, carried on the chylomicrons, leads to the hydrolysis of triacylglycerols carried in the chylomicrons, resulting in the formation of free fatty acids (Feingold, 2022). In addition to LPL activation, major functions of apolipoproteins include that of a structural component, binding to lipoprotein receptors, chaperoning the formation of lipoproteins, and assisting as inhibitors or activators of enzymes involved in the metabolism of lipoproteins. For example, Apo B-48 is the major structural protein of chylomicrons and chylomicron remnants, while Apo-E swaps between various lipoprotein particles and is connected with chylomicron remnants, chylomicrons, VLDL, IDL, and HDL particle subgroups. The free fatty acids released from the chylomicrons can subsequently be taken up by adjacent muscle cells and adipocytes for energy production or storage. The uptake of fatty acids is facilitated by CD36, in addition to fatty acid transport proteins (FATPs) (Drover et al., 2005; Feingold, 2022; Wolska and Remaley, 2021). CD36, which can be found on the cytoplasmic membrane of a variety of epithelial cells as well as mononuclear cells, platelets, and adipocytes, functions as a nonspecific scavenger receptor (Coburn and Abumrad, 2003). Among a variety of other functions, e.g., angiogenesis and cellular adhesion, CD36, also known as fatty acid (FA) translocase (FAT), is involved in lipoprotein binding, endocytosis as well as long-chain FAs uptake (Tandon et al., 1989; Endemann et al., 1993; Dawson et al., 1997; Coburn et al., 2000).

### Pathologic changes

Genetic predisposition for hypertriacylglycerolemia in individuals can be due to mutations in genes associated with Apo C-II, GPIHPB1, lipase maturation factor 1a, and loss of function mutations in LPL and Apo A-V, which plays an important role in LPL activity, Apo C-II, GPIHPB1, and lipase maturation factor 1 can result in marked hypertriacylglycerolemia. In addition, loss of function mutations in Apo C-III, which inhibits LPL activity, are associated with increases in LPL activity and decreases in plasma triacylglycerols levels. Similarly, angiopoietin–like protein 3 and 4, which target LPL for inactivation, regulate LPL activity. Loss of function mutations in these proteins are also associated with decreases in plasma triacylglycerols levels. Finally, the expression of LPL by muscle cells and adipocytes is regulated by hormones, particularly insulin, nutritional status, and inflammation (Wolska and Remaley, 2021; Feingold, 2022).

Human gene polymorphisms in *APOA5* have been reported to be associated with high and low triacylglycerol concentrations. Triacylglycerols deficiency results in reduced LPL activity and type V dyslipidemia. This possibly occurs through Apo A-V’s effect on the lipolysis of triacylglycerol-rich lipoproteins, through its binding to lipoprotein, endothelial proteoglycans and LPL resulting in stabilization of the lipolytic machinery.

### Chylomicrons and high-density lipoprotein (HDL) formation and maturation

**FIGURE 2:**
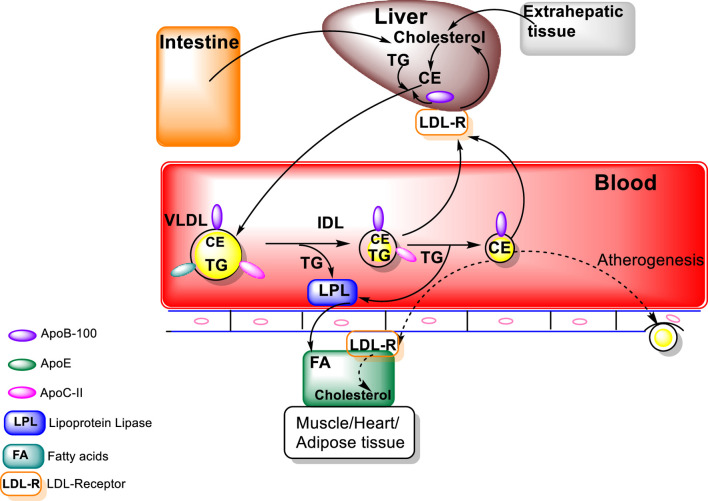
Endogenous Lipoprotein Pathway (figure modified from “Introduction to Lipids and lipoproteins”, K.R.Feingold, https://www.ncbi.nlm.nih.gov/books/NBK305896/).

Metabolism of the triacylglycerols carried in the chylomicrons results in a marked decrease in the size of these particles leading to the formation of chylomicron remnants, which are enriched in cholesterol esters and acquire Apo E (Figure 1). As these particles decrease in size, phospholipids and apolipoproteins (Apo A and C) on the surface of the chylomicron remnants are transferred to other lipoproteins, mainly HDL. The transfer of Apo C-II from chylomicrons to HDL decreases the ability of LPL to further breakdown triacylglycerols. These chylomicron remnants are cleared from the circulation by the liver (Wolska and Remaley, 2021; Feingold, 2022). LPL hydrolyses the TG to FA that can be taken by cells, thus, transforming the chylomicrons and VLDL to smaller lipoprotein particles, chylomicron remnants and intermediate density LPs (IDL) (Figure 2). The major structural component of VLDL, IDL, and LDL is Apo B-100 which is manufactured in the liver. VLDL, IDL, and LDL each contain a single molecule of Apo B-100 per moiety. Apo B-100 functions as an attachment ligand that binds to the LDL receptor; hence, it is of relative importance in the clearance of lipoprotein particles. The presence of elevated levels of Apo B-100 are associated with increased risk of atherosclerosis. Inversely related to atherosclerotic risk is the level of mature HDL particles. Several steps are required to generate mature HDL particles. The cholesterol that is effluxed from cells to HDL is free cholesterol that is localized on the surface of HDL particles.

To form mature large spherical HDL particles with a core of cholesterol esters, the free cholesterol must be esterified in order to be transferred from cells to the HDL particles (Wolska and Remaley, 2021; Feingold, 2022). The major structural protein of HDL is Apo A-I, which also plays a role in HDL interaction with ATP-binding cassette protein A1 (ABCA1), ABCG1, and class B, type I scavenger receptor (SR-B1) (Oram and Lawn. 2001; Van Eck et al., 2005). In addition, Apo A-I also serves as a lecithin: cholesterol acyltransferase (LCAT) activator. LCAT is an enzyme that converts free cholesterol into cholesteryl ester (CE). High levels of Apo A-I are associated with a lowered risk for atherosclerosis.

### Lecithin–cholesterol acyltransferase (LCAT)

**FIGURE 3:**
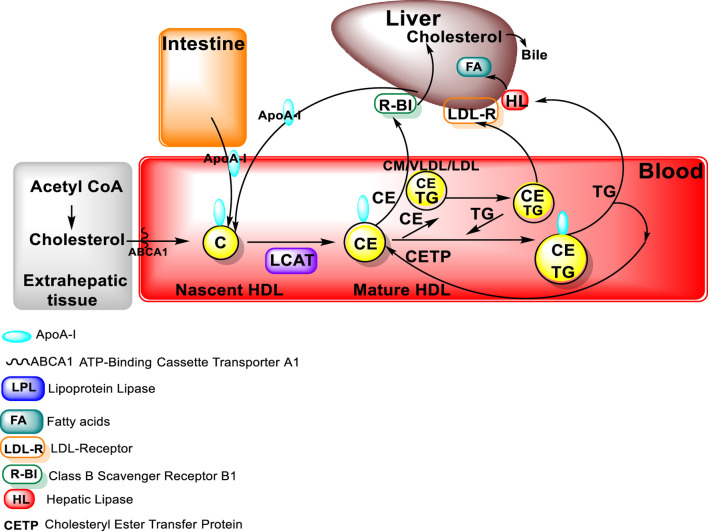
LCAT in HDL metabolism (figure modified from “Introduction to Lipids and lipoproteins”, K.R.Feingold, https://www.ncbi.nlm.nih.gov/books/NBK305896/).

As indicated above, LCAT activity is required for the formation of large HDL particles (Figure 3). LCAT is an HDL-associated enzyme that catalyzes the transfer of a fatty acid from phospholipids to free cholesterol, resulting in the formation of these cholesterol esters. The cholesterol ester formed is then able to move from the surface of the HDL particle to the core of the HDL particle (Feingold, 2022). The esterification process of LCAT is facilitated by Apo A-I, an LCAT activator**.** LCAT deficiency in humans results in decreased HDL cholesterol, Apo A-I levels, and a higher percentage of small HDL particles. Pathologically, LCAT deficiency can result in impaired vision, due to cholesterol corneal opacities, as well as anemia and renal damage as occurs in familial lecithin:cholesterol acyltransferase deficiency (FLD) disease, an autosomal recessive disorder (Feingold, 2022).

## Current therapeutic approaches for activating LCAT and LPL in host disorders

### Defects in LCAT

LCAT gene polymorphisms play a role in several physiologic disorders, with138 mutations of the LCAT gene reported (Mehta et al., 2021). Genetic LCAT deficiency is a rare autosomal recessive disorder with an incidence of less than 1 in 200,000, although the frequency is likely to be higher due to misdiagnosis, or under diagnosis. Mutations in the LCAT protein, which causes a loss, or reduction, of LCAT activity, are responsible for familial LCAT deficiency (FLD), or fish eye disease (FED). FLD is characterized by the absence of LCAT activity for both HDL and LDL. FED is a partial LCAT deficiency characterized by the absence of LCAT activity toward HDL only. Originally, it was thought the different manifestations of FLD and FED may be due to the residual amount of LCAT activity present on either HDL or LDL particles. It is now understood that these two disorders represent a continuum of LCAT deficiency with FLD patients having a more profound decrease in total LCAT activity. Clinically, the absence of proteinuria and other signs of renal damage are often used to distinguish between these two disorders, although the development of renal disease often takes decades to manifest, and thus, the prognosis of a patient with a new LCAT mutation diagnosis may remain uncertain (Wolska and Remaley, 2021; Feingold, 2022).

FLD is characterized by extremely low or absent HDL, mild to moderate hypertriacylglycerolemia, the development of cloudy cornea in the teenage years, followed by early asymptomatic proteinuria. Normochromic anemia often then develops over the next decade, but it is typically mild. Proteinuria typically steadily progresses to nephrotic syndrome, resulting in end stage renal disease in the fourth or fifth decade of life. The clinical features of FED are also extremely low or absent HDL, and development of cloudy corneas in the teenage years, but the absence of any significant proteinuria and renal disease. Heterozygotes for FLD or FED have no outward clinical symptoms and only have a mildly reduced HDL-C (20–30 mg/dL, 0.52–0.78 mmole/L), but may be at an increased risk for cardiovascular disease (Wolska and Remaley, 2021; Feingold, 2022).

Another genetic defect in LCAT can result in the formation of Lipoprotein X (LpX), an abnormal cholesterol and phospholipid rich lipoprotein particle that is poor in neutral lipids (cholesterol esters and triacylglycerols). Unlike normal lipoproteins, which have a micellar-like arrangement of a single layer of phospholipids surrounding a hydrophobic core of cholesterol ester and triacylglycerols, LpX has a vesicular structure. Because of the high ratio of amphipathic surface lipids (unesterified cholesterol and phospholipids) to neutral core lipids, LpX forms a bilayer of phospholipids, or even a multilamellar phospholipid arrangement, which results in its “onion-like” appearance on electron microscopy. LpX particles are heterogeneous in size (30–100 nm) and can have a density between that of LDL and VLDL. The genesis of LpX in patients with FLD is not known, but the low level of plasma cholesterol esters most likely contributes to its formation in this disorder. In addition, it is likely that the presence of LpX particles is a major precipitating factor in the development of renal disease in FLD patients (Wolska and Remaley, 2021; Feingold, 2022).

Clinically, in certain instances LCAT inhibition is appropriate, such as in lysosomal acid lipase (LAL) deficiency, a rare autosomal recessive disorder. Children born with mutations in both copies of the gene for the lysosomal acid lipase enzyme are unable to process cholesterol esters and triacylglycerols resulting in liver fibrosis, cirrhosis, and liver failure. The most severe form of LAL deficiency, Wolman’s disease, is fatal in the first year of life due to the rapid buildup of cholesteryl esters and triacylglycerols in the liver, gut, and blood vessels. A more prevalent and benign form of LAL-deficiency is cholesterol ester storage disorder (CESD). Individuals affected with this disorder have only a partial loss of LAL activity. Clinically CESD patients are diagnosed with hepatomegaly in early childhood and often go on to develop liver cirrhosis and eventual liver failure. In addition, individuals with CESD are also at increased risk for developing premature atherosclerosis, due to hypercholesterolemia, and increased atheroma foam cell formation. LCAT inhibition prevents the formation of cholesterol esters and would likely extend the life of patients suffering from LAL deficiency. While inhibiting LCAT may lead to the problems associated with FLD, due to age of onset with the symptoms of FLD not manifesting until middle age, LCAT inhibition may be a reasonable course of treatment management for LAL deficiency patients (Wolska and Remaley, 2021; Feingold, 2022).

### LCAT modulators in drug discovery

Since LCAT activators are useful for treating atherosclerosis, FLD, and FED, the focus of drug development is on the LCAT activators. Recombinant human LCAT (rhLCAT), which raises HDL-C and increases cholesterol efflux, was shown to be safe in phase I study (Shamburek et al., 2016a; Shamburek et al., 2016b), followed by phase II trials for CHD (clinicaltrials.gov, NCT02601560, NCT03578809, NCT03773172; Reyes-Soffer. et al. , 2023). The rhLCAT has also been tested in enzyme replacement therapy for several patients with FLD with encouraging results (Shamburek et al., 2016a; Reyes-Soffer et al., 2023). Further studies show (Reyes-Soffer et al., 2023) that an acute increase in LCAT activity can lead to a greater flux of cholesteryl esters (CE), resulting in alteration in production and clearance of the principal HDL proteins but exclusive of affecting APOB100-lipoprotein metabolism. Long-term elevations of LCAT might, therefore, have beneficial effects on total body cholesterol balance and atherogenesis (Reyes-Soffer et al., 2023). However, small molecule activators would be less expensive, and easier to administer than proteins (e.g., rhLCAT). Towards this end, several small molecules that stimulate the activity of LCAT have been identified.

**FIGURE 4:**
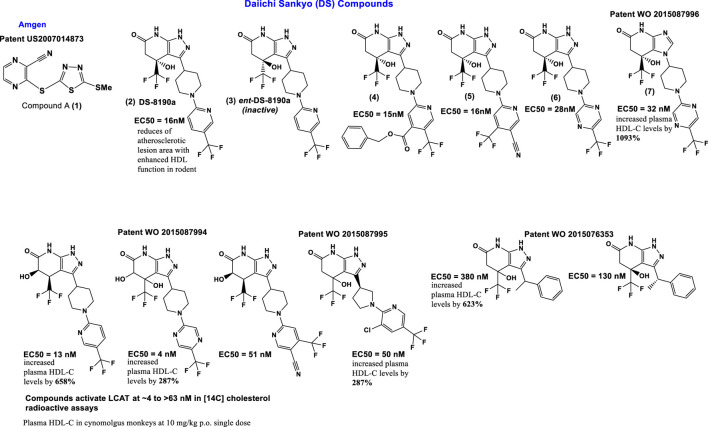
Scaffolds of the small molecules currently identified as LCAT activators.

Compound A (**1**) (3-(5-(ethylthio) pyrazine-2-carbonitrile; Amgen); Figure 4) binds covalently and irreversibly to LCAT by alkylating Cys31 near the active site of LCAT. It increased LCAT activity in three of nine LCAT mutation subsets to levels comparable to FLD heterozygotes (Freeman et al., 2017). Compound A can activate plasma LCAT in the EC50 range of 1–10 μmol/L, for the 4 species tested (C57Bl/6 mouse, hamster, rhesus monkey, and human) (Chen et al., 2012; Kayser et al., 2013; Saleheen et al., 2015; Freeman et al., 2017).

Other sulfhydryl-reactive compounds based on monocyclic β-lactams have also been shown to activate LCAT (Freeman et al., 2017). Although highlighting the promise of these type of LCAT-activating molecules, they most likely would also have off-target effects. Daiichi Sankyo (DS) has reported a new class of reversible small molecule activators (Figure 4) that have the ability to activate LCAT isolated from human plasma (Kobayashi et al., 2015a; Kobayashi et al., 2015b; Onoda et al., 2015; Kobayashi et al., 2016). Furthermore, these activators increased HDL-C up to 1000-fold when tested in a primate model (cynomolgus monkeys) using orally administration (Onoda et al., 2015).

Additional investigation into the activity of the DS LCAT activators, more specifically **DS-8190a** (**2,** Figure 4), demonstrated its ability to prevent the progression of plaque accumulation in atherosclerosis models, and the selectivity of its binding to LCAT (Sasaki et al., 2021). It is interesting to note that the enantiomer of compound **DS-8190a** (**3**, Figure 4) is inactive, with no effect on the quantity of LCAT (Sasaki et al., 2021). The DS type of activators bind in a pocket formed exclusively by the membrane-binding domain (MBD) of LCAT but does not influence affinity of LCAT for HDL (Manthei et al., 2018). Estimation of the binding mode of biotinylated active **DS-8190a** (**3**, Figure 4) found that a binding site experimentally identified by photoaffinity labeling is consistent with the *in silico* simulated model (Sasaki et al., 2021) and its analogues to the binding site for these compounds determined earlier (Manthei et al., 2018).

Piperidinylpyrazolopyridine-based DS activators (e.g., **2**, **4**–**6**, Figure 4) and related activators (Figure 4) stimulate and stabilize LCAT. The piperidinylpyrazolopyridine activators bind exclusively to the membrane-binding domain (MBD). Functional studies indicate that these compounds do not modulate the affinity of LCAT for HDL, but instead stabilize residues in the MBD and facilitate channeling of substrates into the active site. The increased activity of an FLD variant by the DS activators demonstrates that compounds targeting the MBD have therapeutic potential (Manthei et al., 2018; Sasaki et al., 2021). Three DS compounds, compounds **5**, **6** and **7** (Figure 4) have been chosen for determining DS compounds’ mechanism of action (Manthei et al., 2018). The pyrazole ring forms hydrogen bonds with the backbone carbonyl of Met49 and amide of Tyr51 in the binding pocket of the LCAT lid (Manthei et al., 2018). It is interesting to note that while the exchange of pyrazole (compound **6**, Figure 4) to imidazole (compound **7**, Figure 4) eliminates the hydrogen bond with Met49, only a minimal change in EC50 (280 and 320 nM for **6** and **7**, respectively) and no change in the maximum response were observed. These piperidinylpyrazolopyridine-containing compounds (such as **5**, **6** and **7** Figure 4) also recover acyltransferase activity when used in variants of Arg244 within the lid, highlighting the promise of compounds that target the MBD for many missense FLD variants (Manthei et al., 2018).

The potency and efficacy of the DS LCAT activators appear to be highly dependent on the presence and chirality of the hydroxyl at the C4 position, as well as the presence of a pyrazine ring system which most likely is responsible for interactions with hydrophobic substrates (Manthei et al., 2018). The “lid” which contains positions mutated in FLD, undergoes a large conformational change from that observed in inactive LCAT structures. Arg244, which interacts with backbone carbonyls of Leu223 and Leu285 in DNDC-IDFP-1, and with the side chain of Asp335 in the lid closed state, is frequently mutated, thus affecting lid closure Plasma screening studies further showed R244G R244H R244C and R244L mutations in LCAT (Vrabec et al., 1988; McLean, 1992; Pisciotta et al., 2005; Charlton-Menys et al., 2007; Strøm et al., 2011; Sampaio et al., 2017; Castro-Ferreira et al., 2018). Of the human variants tested, LCAT-R244G, isolated from an individual having two identical alleles, was shown to form unique interactions with LCAT (active and inactive) and its side chains, with a loss of ∼85% LCAT-WT activity. In contrast, R244G and R244H heterozygotes exhibited a loss of ∼20% and ∼50% LCAT-WT activity, respectively (Vrabec et al., 1988; Pisciotta et al., 2005). These findings together support the hypothesis that Arg244 is a residue important for LCAT activity.

A high-throughput Daichii-Sankyo screening campaign identified a noncovalent LCAT activator as a hit compound and its derivatization led to novel potent LCAT activators, including **DS-8190a** (compound **2**) shown here. **DS-8190a** is an orally active representative of non-covalent DS LCAT activators, that dose dependently increased LCAT activity (2.0-fold in 3 mg/kg group on day 7) in cynomolgus monkeys, resulting in HDL cholesterol elevation without drastic changes of non-HDL cholesterol (Sasaki et al., 2021). **DS-8190a** binds to the same lid region of LCAT (Sasaki et al., 2021), as identified earlier by Manthei et al. (2018). Compound A is a reference compound forming a co-crystal with human LCAT (Sasaki et al., 2021) that together with compound **6** and **7** is used in the determination of DS-compounds’ mechanism (Manthei et al., 2018).

**FIGURE 5:**
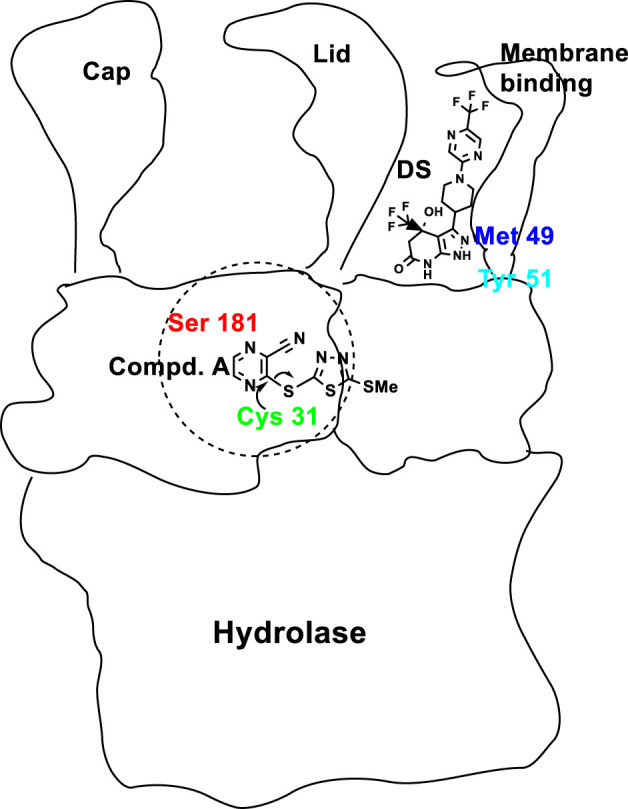
Cartoon of LCAT domains and the various areas of binding of Compound A (**1**, Figure 4), and DS compounds, e.g., **6**, (Figure 4), with Cys31 near the active site and Met49/Tyr51 of the lid, respectively. The active sites of many lipases are contained in the N-terminal domain and controlled by a so-called lid. The catalytic triad, Ser-Asp-His, is at the bottom of lid’s crevice (Berton et al., 2007). The lid domain covers the active site to control both enzyme activation and substrate specificity.

Active site Ser181 as part of the catalytic triad of LCAT (Ser181, Asp345, His377), acts as the nucleophile in formation of CE (Ahsan et al., 2014). Compound A, a Michael acceptor, binds covalently (and irreversibly) to Cys31 (Freeman et al., 2017) (Figure 5). Critical for LCAT activity are the six cysteines, four in disulfide bonds (Cys50-Cys74 and Cys313-Cys356) (Glukhova et al., 2015). The remaining two free cysteines (Cys31 and Cys184) are located close to the active site, with Cys31’s backbone amide forming a portion of the oxyanion hole (Holleboom et al., 2011; Glukhova et al., 2015). DS compounds interact noncovalently with the LCAT’s membrane-binding domain, causing conformational changes, as confirmed by X-ray crystallography**,** leading to opening of the lid (Manthei et al., 2018; Sasaki et al., 2021) (Figure 5).

*Defects in LPL* are a cause of familial chylomicronemia syndrome (or type I hyperlipoproteinemia) and of a form of deficiency characterized by hypertriacylglycerolemia (Burnett et al., 1999). Familial chylomicronemia (FCS) is a recessive disorder usually manifesting in childhood (Evans and Kastelein, 2003). It is a rare autosomal recessive disorder with an incidence of ∼1:1,000,000 caused by mutations in lipoprotein lipase, resulting in accumulation of chylomicrons in plasma and hypertriacylglycerolemia (Rahalkar et al., 2009). Elevated triacylglycerols cause several complications in patients, the most serious being episodes of acute pancreatitis (Rahalkar et al., 2009). Less often, a neonatal onset of LPL-deficiency is diagnosed based on routine blood testing (Akesson et al., 2016). Even a small amount of dietary fat can make someone with FCS symptomatic. In addition to familial chylomicronemia syndrome, more than 200 mutations were reported in patients with LPL deficiency (Ranganathan et al., 2012; Wang et al., 2013; Kolarova et al., 2014; Wu et al., 2021). To date, the pharmacologic treatment for affected patients is Evinacumab, a human monoclonal antibody approved by the U.S. Food and Drug Administration (FDA) in 2021 as an add-on treatment for patients with homozygous familial hypercholesterolemia (Witztum et al., 2019). The management options for the latter primarily include adoption of an extremely restricted, very-low-fat diet, along with avoidance of certain medications and alcohol. Volanesorsen, an antisense oligonucleotide, has been recently approved by the European Medicines Agency as an adjunct to diet in adult FCS patients with an inadequate response to TG-lowering therapy (Kolovou et al., 2022).

*LPL is an interesting target for drug discovery* because of its central role in TG metabolism (Geldenhuys et al., 2017). Increased LPL activity may compensate for deleterious alleles that increase CVD risk due to hypertriacylglycerolemia and adverse central nervous system events, the latter of which is slowly being unraveled. Immunostaining for LPL has been detected in the neurons, astrocytes, microglia, and oligodendroglia throughout the central nervous system (Wang et al., 2011). LPL can mediate the uptake of TGs and their subsequent incorporation into cellular lipids in cultured brain cells. LPL plays a role in the differentiation of neurons *in vivo*, and regeneration of neuronal processes. LPL has been shown to regulate energy balance and bodyweight in mice because a neuron-specific deletion of LPL exhibits obese phenotype in mice fed standard chow (Gong et al., 2013). Further, mouse studies indicate that LPL functions in Alzheimer’s disease (AD) pathology. LPL is found in AD amyloid plaques, along with at least six other LRP-binding proteins (Rebeck et al., 1995). LPL in AD is consistent with the proposed protective functions of LPL in brain injury models, acting to modify the synaptic loss/remodeling process and adult neurogenesis (Blain and Poirier, 2004). Most recently, LPL has also been shown to bind to amyloid beta protein (Aβ), the peptide which comprises AD amyloid plaques, and promote cell-surface association and uptake of Aβ in mouse primary astrocytes, providing an alternative mechanism of how LPL might play a role in AD (Geldenhuys et al., 2017).

Although LPL has been studied intensively for >60 years, its structure had not been determined until recently, likely stemming from the susceptibility of LPL’s hydrolase domain to unfolding. The discovery that the protein GPIHBP1 stabilizes LPL structure and activity allowed for the crystal structure of an LPL–GPIHBP1 complex to be determined. GPIHBP1 is the endothelial cell partner protein of LPL, and is a small, cysteine-rich protein binding to LPL through hydrophobic interactions. GPIHBP1 is not involved in lipid binding; rather, its functions are to chaperone LPL across endothelial cells and stabilize LPL structure (Birrane et al., 2019). LPL is organized into two structurally distinct domains. The bigger N-terminal domain contains a binding site for heparin and the binding site of apolipoprotein C-II (APOC2) (Birrane et al., 2019). Also housed in the N terminus is the catalytic site of the enzyme comprising the triad: Ser132, Asp156 and His241 (Birrane et al., 2019). The smaller C-terminal domain has been shown to be important for binding lipoproteins. The active site of the LPL is covered by a “lid”; as seen in other homologs such as hepatic lipase (HL) and pancreatic lipase (PL) (Mysling et al., 2016). The lid is postulated to have an impact on the substrate specificity of the lipase gene family and is essential for interaction with the lipid substrate (Young and Zechner, 2013; Birrane et al., 2019). Open and closed “lid” conformations can assist downstream in the functional activation of hydrolysis. Lipases achieve an open state by undergoing interfacial activation, which occurs when the lipase associates with a nonpolar-aqueous interface (van Tilbeurgh et al., 1993). The combination of the lid peptide and hydrophobic pocket is thought to provide mammalian lipases with substrate selectivity. Certain fungal lipases, specifically that from *Candida rugosa,* are exceptions to the functionality described above (Grochulski et al., 1994) since it uses a channel rather than a pocket for substrate binding. Subsequent studies show that when mutations are made in the channel the substrate specificity is changed (Kingsley and Lill, 2015). Furthermore, it has been recently reported that adjacent to the active site mammalian LPL has in its dimeric form a hydrophobic pore spanning the N-terminal domain. This indicates that this pore may play a role in acyl chain hydrolysis (Gunn et al., 2020; Gunn and Neher, 2023). This ground-breaking finding changes the currently prevailing theory that a displaced lid peptide, which subsequently exposes the hydrophobic pocket that surrounds the active site, is required for an open lipase conformation. Mechanistically, post-lid opening the substrate enters the lipase active site, is hydrolyzed and subsequently released bidirectionally. However, based on the new findings, the model for lipid hydrolysis that has been proposed is one in which the free fatty acid product is transported through the active site pore in a unidirectional “one-way” manner. This provides more substrate selectivity by the pore. Unfortunately, due to the inherent difficulty in analyzing lipase structures in the presence of substrate this has yet to be proven although there is the evolutionary suggestion that there is a high degree of conservation of LPL structure across other human lipases this has yet to be proven (Gunn and Neher, 2023).

### LPL modulators in drug discovery

Glybera (alipogene tiparvovec), a gene therapy product that replaces the LPL gene is available in Europe from uniQure (www.uniqure.com) for the treatment of patients diagno244sed with a genetic deficiency in familial lipoprotein lipase disease (LPLD). A bihelical amphipathic peptide (C-II-a) that contains an amphipathic helix (18A) based on apoC-II, which promotes cholesterol efflux and lipolysis, has been prepared and is being tested *in vivo* by A.T. Remaley and co-workers (Amar et al., 2015; Wolska et al., 2020). C-II-a is expected to be useful for the treatment of apoC-II deficiency, as well as other forms of hypertriacylglycerolemia.

**FIGURE 6:**
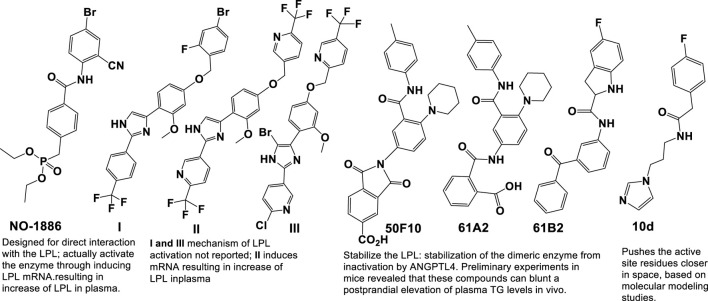
Scaffolds of the small molecules currently identified as LPL activators.

A large gap in the LPL field is the lack of clinically used small molecule drugs to modulate LPL activity. The structures of several small molecules identified as LPL activators are given below (Figure 6). Previous attempts to modulate LPL activity using small molecules produced highly significant effects in several animal models of hyperlipidemia. **NO-1886** (generic name: Ibrolipim, Otsuka Pharmaceutical Factory; Figure 6) was shown to significantly stimulate LPL activity, lower plasma triacylglycerols, as well as elevate the levels of HDL-C (Tsutsumi et al., 1993). Mechanistically **NO-1886** increases LPL mRNA, rather than directly affecting the enzyme, thereby increasing post-heparin LPL mass (Tsutsumi et al., 1993). In streptozotocin (STZ) treated diabetic rats, **NO-1886** increased LPL activity 59% over the control. Long term **NO-1886** administration to rats and rabbits, with high-cholesterol feed-induced atherosclerosis, significantly inhibited development of atherosclerotic lesions in coronary arteries (Tsutsumi et al., 1993). However, the compound had adverse species-specific side-effect on adrenal cortex steroidogenesis leading to hypertrophy of adrenal glands in rats and dogs, although this effect was not observed in monkeys. Therefore, due to potential side effects further development of **NO-1886** for use in humans was halted. Additional efforts to enhance LPL activity, by inhibiting ANGPTL3/8 complex with monoclonal Ab, showed dose dependent reduction in TG, LDL-C, non-HDL-C in phase 1 trials (Gaudet et al., 2022).

Consequently, three compounds based on phenylimidazole scaffold (**I**, **II**, and **III**, Figure 6) were developed (Shibutani et al., 2010; Iwata et al., 2017; Iwata et al., 2018). Compound **I** (Figure 6) showed 23% LPL activation at 10 mM in a human skeletal muscle myoblasts cell assay (Shibutani et al., 2010). Compound **II** (Figure 6) demonstrated 2.97% potentiation effect of LPL mRNA (Iwata et al., 2017). Compound **III** (Figure 6) is described as an LPL activator and effective in the prevention and treatment of hyperlipidemia and obesity (Iwata et al., 2018). One compound, **10d** (Figure 6), exhibited potent LPL activation, twofold that measured for **NO-1886** (Geldenhuys et al., 2014). Compound **10d** is presumed to activate LPL directly, by pushing the active site residues closer in space, based on molecular modeling studies (Geldenhuys et al., 2014).

N-phenylphthalimide derivative **50F10** (Figure 6) has recently been identified in a small molecule screen designed to select compounds that protect LPL against inhibition by ANGPTL4 (Larsson et al., 2014). Further development based on the original scaffold led to identification of carboxamides **61A** and **61B**, (Figure 6), with increased stability in plasma, as compared to **50F10** that exhibited potent activity in primary and secondary screens using different LPL substrates (Larsson et al., 2014). Mechanistic studies showed that **50F10**, **61A** and **61B** (Figure 6) stabilize the active homodimer structure of LPL and prevent its conversion to inactive monomers in the presence of ANGPTL4 (Caraballo et al., 2015).

## Modulators of host LCAT activity by bacteria, viruses and fungi


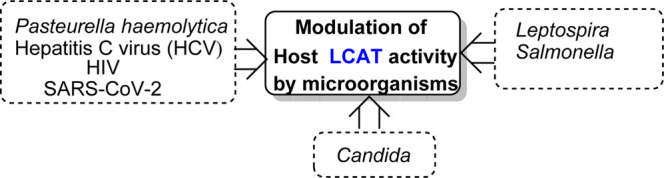


Reports addressing the changes in plasma lipids by infections began to appear in the literature approximately six decades ago, around the same time as LCAT was first described (Glomset, 1962; Glomset, 1968). These lipid changes have been associated with bacterial, viral, and protozoal infections, as well as cancer (Grossberg and O’Leary, 1965; Farshtchi and Lewis, 1968; Fiser et al., 1972; Sakaguchi and Sakaguchi, 1979; Coombes et al., 1980; Rouzer and Cerami, 1980; Sakaguchi, 1982; Budd and Ginsberg, 1986; Auerbach and Parks, 1989; Yamamoto and Katoh, 2000).

**FIGURE 7:**
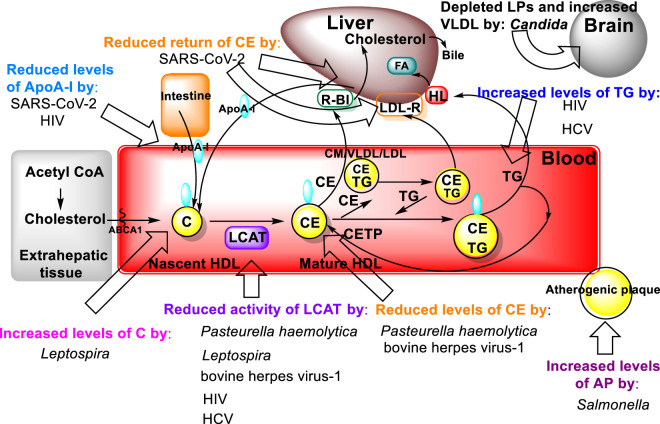
Bacterial molecular targets in the lipid metabolic pathways of the host (Figure 3) and their effects on lipid metabolism.

A typical host response to infection is an elevation in plasma triacylglycerols concomitant to a decrease in total plasma and HDL cholesterol concentrations (Sakaguchi, 1982; Budd and Ginsberg, 1986; Yamamoto and Katoh, 2000) (Figure 7). The acquired hypocholesterolemia that accompanies infections, or malignant illness, has even been suggested as an indicator of poor prognostic outcome, e.g., in rat models and clinically in human liver cancer, where it is often lower than normal tissue (Oster et al., 1981; Pattanayak, Sunita, and Mazumder, 2014; Ouyang et al., 2020). Conversely, high LCAT expression is associated with an improved prognosis for liver cancer patients (Long et al., 2019). Additional common changes that occur in response to infection are increased Very Low-Density Lipoprotein, made of 60% tris (VLDL), and decreased HDL cholesterol concentrations (Budd and Ginsberg, 1986). It has been demonstrated that the serum activity of LCAT, and the concentration of cholesteryl esters, both constituents of the HDL fraction, are reduced in calves inoculated with *Pasteurella haemolytica* and bovine herpes virus-1, the two major pathogens of bovine pneumonia (Yamamoto and Katoh, 2000).

Another bacterial genus, *Leptospira*, whose species are the causative agents of the zoonotic disease leptospirosis, exhibits lipid modulation in infected individuals (Silva et al., 2020). In addition to several alterations in both plasma lipids and erythrocyte membranes, the LCAT fractional activity was 3.6 times lower in individuals with leptospirosis than in the healthy individuals, which also resulted in an increased free plasma cholesterol levels in these patients (Silva et al., 2020). Mechanistically, elevated bacterial lipopolysaccharide (LPS) binding LP complexes using a *Salmonella* model (*Salmonella minnesota* R595 ^125^I-labeled LPS) to evaluate initiation of atherogenic plaque formation showed that LPS-LP fractions in complex formation as an risk factor for initiating atherosclerosis in hypercholesterolemia (Schwartz and Dushkin, 2002). Furthermore, two other major components of dyslipidemia, namely, hypertriacylglycerolemia and decreased levels of HDL, which also contributes to increased atherosclerotic risk, have been observed in individuals with HIV (Mirajkar et al., 2017). These dyslipidemic changes are attributed to decreased LCAT activity together with a decline in apo A-I levels leading to a reduction in the reverse cholesterol transport (Mirajkar et al., 2017). Similar findings were reported for hepatitis C virus (HCV) infections (Blaising and Pécheur, 2013) (Figure 7).

HCV is a major human pathogen in which lipids are an essential part of its pathology. A common feature of chronic hepatitis C is steatosis, characterized by excessive accumulation of triacylglycerols and lipid content in the liver. Thus, HCV infection appears to be closely connected to host cell lipid metabolism, from viral cell entry, through viral RNA replication to viral particle production and formation/assembly (Blaising and Pécheur, 2013).

During SARS-CoV-2 disease a temporary disturbance of lipid metabolism occurs that is principally due to impairment of HDL function (Sorokin et al., 2020; Feingold, 2023). The infection of pulmonary tissue by SARS-CoV-2 results in activation of alveolar macrophages and an ensuing cytokine storm, as a result of the release of inflammatory mediators. The activation of immune system mediators and uncontrolled inflammation leads to the impairment of HDL lipoprotein function by reducing the concentration of apolipoprotein AI (ApoA-I), apolipoprotein E (ApoE) and increasing the concentration of serum amyloid A (SAA). These lipid changes reduce the anti-inflammatory, antioxidant, and immunomodulatory properties of HDL lipoproteins. Oxidized HDL and LDL lipoproteins (oxLDL and oxHDL) are potent activators of the oxidized LDL scavenge receptor (LOX-1), causing further inflammation and tissue damage (Sorokin et al., 2020). The increased percentage of oxidized oxLDL and oxHDL lipoproteins in turn leads to impairment of cholesterol re-transport that is characterized by an insufficient interaction of ApoA-I with the ATP-binding cassette transporter (ABCA1) on macrophages and decreased esterification of cholesterol by lecithin cholesterol acyltransferase (LCAT) (Oram and Lawn. 2001).

The pathophysiological effect of this is a reduced return of cholesterol esters to the liver immediately after interaction with hepatic SR-B1, or indirectly after transfer to LDL by cholesterol ester transfer protein (CETP) and uptake by hepatic LDL receptors (LDL-R). Low concentrations of ApoE and apolipoprotein C-III (ApoC-III) on HDL reduce the activity of lipoprotein lipase (LPL), which then leads to the accumulation of very low-density lipoproteins (VLDL) and triacylglycerols. It is also worth mentioning that oxidized phospholipids in LDL lipoproteins are recognized as danger-associated molecular patterns (DAMPs), which cause inflammasome stimulation, impaired vascular endothelial cell function and atherosclerotic progression. The effects of the interaction of oxidized LDL (oxLDL) and LOX-1 (accumulation of oxLDL inside the cells) also contribute to the accelerated atherosclerosis progression (Sorokin et al., 2020).

In addition to the aforementioned observations of lipid modulation caused by pathogens that occur in plasma and internal organs, such as the liver, the colonization of the brain by fungal pathogens, mainly *C. albicans,* and the lipid modification there has been proposed as a model for Alzheimer’s disease (Parady, 2018). It is well established that HDL slows progression of CVD by inhibiting cytokine induced expression of adhesion molecules that enable leukocytes to adhere to the endothelium (Cockerill et al., 1995). The latter process leads to increasing adhesion of *Candida albicans*. The role of lipid dysmetabolism in susceptibility to candidiasis is evidenced both *in vitro* and in the ApoE deficient mouse model. In culture, when *Candida* is grown with lipids it increases its expression of virulence factors and rate of replication (Vonk et al., 2004). In the ApoE deficient mouse model, wherein there is depleted lipoproteins and increased VLDL, these mice exhibit increased susceptibility to candidiasis.

### Host LCAT and its modulators as defense against bacterial and viral infection


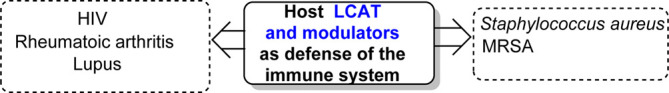


Evidence for the functional inhibition by serum lipoproteins of phenol-soluble modulins (PSM), which are peptides produced by *Staphylococcus aureus* during infection, has been obtained mainly by *in vitro* studies (Surewaard et al., 2012). Among the many virulence factors and immune evasion molecules described PSMs are key virulence factors contributing to the pathogenicity of community-acquired methicillin-resistant *S. aureus* (CA-MRSA) strains (Wang et al., 2007; Li et al., 2009). Recently, *in vivo* studies have demonstrated that HDL particles can efficiently scavenge PSMs, and prevent host cellular damage (Hommes et al., 2021). While *in vitro* serum from both LCAT and ABCA1 knockout mice strains, which are characterized by a near absence of HDL, was shown to fail to protect against PSM-induced neutrophil activation and lysis. Importantly, PSM-induced peritonitis in LCAT^−/−^ mice resulted in increased lysis of resident peritoneal macrophages and enhanced neutrophil recruitment into the peritoneal cavity. Notably, LCAT^−/−^ mice were more likely to succumb to staphylococcal bacteremia in a PSM-dependent manner. Plasma from human homozygous carriers of ABCA1 variants, characterized by very low HDL-cholesterol levels (HDLc), was found to be less protective against PSM-mediated biological functions as compared to healthy humans, therefore lipoproteins present in blood can protect against the key staphylococcal virulence factor PSM (Hommes et al., 2021).

**FIGURE 8:**
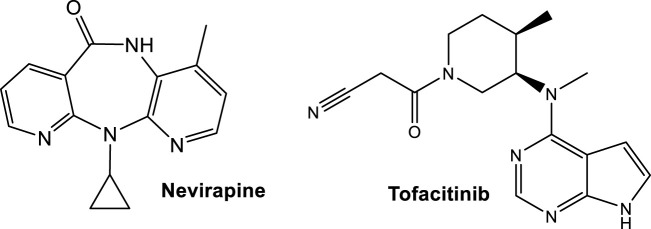
Structures of drugs affecting the cholesterol levels approved by the FDA for CVD-alternative illnesses; Nevirapine as anti-HIV treatment and Tofacitinib for treatment of rheumatoid arthritis, that have a favorable effect in increasing ApoA-I and reducing cholesterol ester catabolism, respectively.

ApoA-I production, which contributes to HDLc increase, occurs in HIV-patients receiving Nevirapine (Figure 8), a nonnucleoside reverse transcriptase inhibitor (NNRTI) (Franssen et al., 2009). The observed increase of apoA-I contributes to a favorable cardiovascular profile, suggesting that NNRTI-based regimens may be helpful in alleviating the changes in HDLc induced by HIV infection. Another component of the antiretroviral cocktail is a protease inhibitor, which is often associated with induction of proatherogenic lipid changes, predominantly increases in LDLc or triacylglycerols (Carr et al., 1998; Franssen et al., 2009).

The suppression of cholesterol levels in patients with active rheumatoid arthritis (RA), like with any inflammation, has also been recognized (Toms et al., 2011). In patients with active RA a dyslipidemia relative to LCAT occurs, where individuals with RA exhibit below normal levels prior to treatment, as compared to healthy controls (Charles-Schoeman et al., 2015). The studies point to the underlying increases in cholesterol ester catabolism as the driving force behind the low cholesterol levels in patients with active RA (Charles-Schoeman et al., 2015). Post-initiation of tofacitinib treatment, both LCAT activity and mass increase (Charles-Schoeman et al., 2015).

Tofacitinib (Figure 8), an FDA approved Janus kinase (JAK) inhibitor, reduces cholesterol ester catabolism, which in turn increases cholesterol levels toward those measured in healthy volunteers, with improved markers in antiatherogenic HDL function (Charles-Schoeman et al., 2015). Tofacitinib, the first tested JAK inhibitor, has a high selectivity for JAK1 and JAK3. It is less effective for the inhibition of JAK2 and has limited action on TYK2 (Chasset et al., 2021). Tofacitinib proved to be safe in systemic lupus erythematosus (SLE), while also improving cardiometabolic and immunologic parameters associated with the premature atherosclerosis in SLE (Hasni et al., 2021; https://classic.clinicaltrials.gov/ct2/show/NCT02535689). In addition, Tofacitinib improved HDLc levels as well as LCAT concentration (Hasni et al., 2021).

## Modulators of host LPL activity by bacteria, viruses and fungi


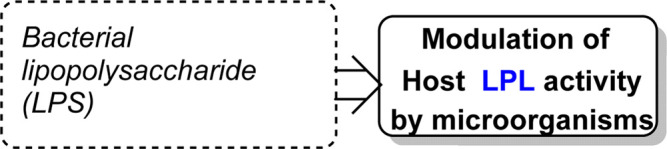


Bacterial lipopolysaccharide (LPS) addition at low concentrations has been demonstrated to dramatically reduce synthesis and secretion of LPL by human macrophages (White et al., 1988). Polymyxin B *in vitro* successfully blocked the decrease in LPL activity, thus confirming that the changes were due to LPS, or a factor stimulated in response to LPS treatment (White et al., 1988).

### Host LPL and its modulators as defense against bacterial, viral and fungal infection


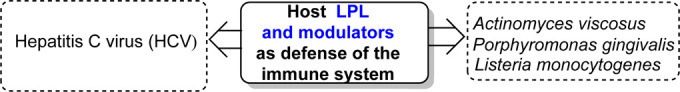


Lipoproteins from Gram-negative oral pathogens including *Actinomyces viscosus* and *Porphyromonas gingivalis* induce inflammatory responses through a Toll-like receptor 2 (TLR2) response that triggers inflammation in the host as well as promotes bacterial persistence (Shimada et al., 2012; Jain et al., 2013). With respect to *P. gingivalis* cells, when they were treated with LPL their ability to activate TLR2 was attenuated (Jain et al., 2013). Similar findings are reported for lipoproteins from the Gram-positive bacterium, *Listeria monocytogenes*, wherein their lipoproteins also exhibit TLR2 agonist activity that has been shown to be sensitive to LPL-mediated inhibition (Machata et al., 2008).

**FIGURE 9:**
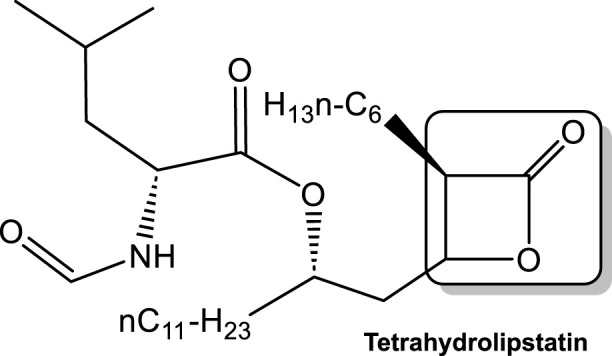
Tetrahydrolipstatin (a representative of statin class of drugs), with the lactone ring as the electrophile responsible for its activity boxed. Lipstatin, a product of *Streptomyces toxytricini* mold inhibits mammalian lipases. Tetrahydrolipstatin (Orlistat), an FDA approved drug synthesized from lipstatin, binds to lipases in the gastrointestinal tract, thus blocking the digestion of dietary triacylglycerols.

The direct protective role against viral invasion of human LPL has also been studied. Several reports with respect to the protective role of LPL against hepatitis C virus (HCV) indicate that this protection is the result of the dependance of this viruses’ lifecycle on lipoprotein metabolism (Thomssen and Bonk, 2002; Andréo et al., 2007; Maillard et al., 2011; Blaising and Pécheur, 2013). During the HCV replicative cycle, the biosynthesis pathway of very low-density lipoprotein (VLDL) is affected. The consequences of this are that HCV virions are associated with triacylglycerol-rich lipoproteins (TRL) in the serum (Thomssen and Bonk, 2002; Andréo et al., 2007; Maillard et al., 2011). LPL was found to have a bridging activity, i.e., mediating the hepatic uptake of chylomicrons and VLDL remnants (Maillard et al., 2011) which is independent of the LPL hydrolytic activity for TRL within chylomicrons and VLDL. Interestingly, *in vitro* the addition of LPL increases HCV binding to hepatoma cells presumably through the effect of LPL on maintaining HCV at the cell surface, thus inhibiting uptake (Maillard et al., 2011). This effect on HCV attachment appears to occur via a LPL bridge between the virus-associated lipoproteins and cell surface heparan sulfate, while simultaneously decreasing infection levels in a manner that is not HCV strain-dependent (Andréo et al., 2007). Further studies showed that lipid droplets may play a role in virion uptake and internalization into hepatoma cells. LPL also appears to efficiently inhibit HCV infection by acting on TRL-associated HCV particles through mechanisms involving both lipolytic and bridging functions, with the bridging function being the main one. However, the lipase inhibitor tetrahydrolipstatin (Figure 9) restored only a minor part of HCV infectivity, suggesting an important role of the LPL bridging function in the inhibition of infection **(** Maillard et al. 2011).

## LCAT/LPL-like enzymes produced by non-mammalian species utilized against the human host

### LCAT-like enzymes (based on their similarities in 3D structure and reaction mechanism to LCAT) produced by bacteria (GCAT) and parasites (*Plasmodium falciparum*)


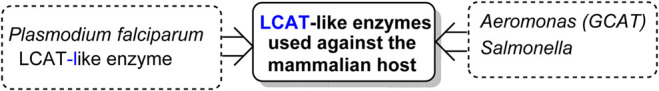


Lipases are typically greatly dissimilar in size and sequence, with only short homologous sequences around the active site. However, X-ray crystalography shows a three-dimensional shared core *α*/*β*-hydrolase fold motif, which is also present in a diverse range of enzymes (Ollis et al., 1992). The first reports for the isolation, purification and activity of microbial LCAT-like enzyme—GCAT (glycerophospholipid:cholesterol acyltransferase) from *Aeromonas* of the bacterial family *Vibrionaceae* appeared in the literature in the early 1980s (Buckley et al., 1982; Buckley, 1982; Buckley, 1983; Buckley et al., 1984). The action of the bacterial acyltransferase, GCAT, is similar in overall reaction mechanism to the LCAT. There are however differences, the principle one being that the relative proportions of cholesterol and cholesteryl ester in normal plasma are near the equilibrium ratio for the reaction carried out by GCAT, which is indicative of the cholesteryl ester formation being very similar to the rate of its hydrolysis. However, the ratio of cholesterol to cholesteryl ester in the plasma of LCAT-deficient patients is substantially reduced in the presence of GCAT (Buckley et al., 1984). In addition, in *Aeromonas salmonicida,* GCAT is complexed with outer membrane lipopolysaccharide forming a lethal toxin for salmon (*Salmo salar* L.). Lee and Ellis (1989), Lee and Ellis (1990).

An LCAT-like enzyme has also been detected in *P. falciparum*, a protozoan, and one of the causative agents of malaria (Ramaprasad et al., 2023). *Plasmodium* replicates intracellularly within erythrocyte vacuoles. The infected red blood cell (RBC) is ruptured upon each round of parasite multiplication in a process known as egress to release a new generation of parasites. Egress is required for the disease to progress. The parasite sends out various molecules to puncture and destroy the membranes of the vacuole, and consequently the RBC, thus securing its egress. Although the identity of these molecules is largely unknown, one of the molecules has been identified by genetic and proteomics approaches as a parasite secreted LCAT that is localized in the vacuole. The importance of LCAT in *Plasmodium* replication is demonstrated by mutant studies that showed parasites lacking LCAT clump together and are also unable to escape infected RBCs resulting in a reduction in the rate of parasitemia (Ramaprasad et al., 2023).

### LPL-like enzymes (based on their similarity in 3D structure and reaction mechanism) produced by bacteria (Lpls) involved in virulence


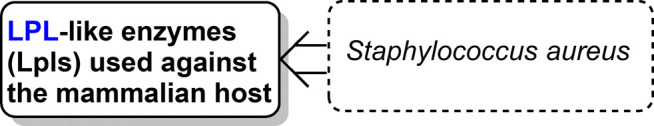


The bacterial molecules that activate the TLR2, discussed above, have been identified as lipoproteins (Jain et al., 2013; Machata et al., 2008). There are now LPLs, similar to the LPL by structure and activity, but have different host targets. In the last decade, it appears that the focus of the Lpls has been on those produced by *S. aureus* affecting several different targets in the host. Lipoproteins represent a major class of surface proteins particularly in Gram-positive bacteria. In *S. aureus* they play a role in both nutritional acquisition and in pathogenicity (Shahmirzadi et al., 2016). In staphylococci, lipoproteins (Lpp) are the main TLR2 agonists and as such they contribute to innate and adaptive immune signaling and modulating the immune response and inflammation (Nguyen and Götz, 2016).

One of the targets of bacterial Lpls has been identified as G2/M phase transition process in host cell division. It has been demonstrated that *S. aureus* LPls delay G2/M phase transitions in HeLa cells (Nguyen et al., 2016). Studies have shown that the *lpl* gene cluster encodes for nine homologous lipoproteins involved in virulence (Nguyen et al., 2015; Nguyen et al., 2015; Shahmirzadi et al., 2016). One of them when tested (Lpl1) was shown to cause G2/M phase transition delay (Nguyen et al., 2016). As discussed earlier, lipid modification of the Lpls is necessary for TLR2-meditated immune stimulation. However, it had not been demonstrated to be necessary for the extension of the G2/M transition delay. Thus, the Lpls’ mediated G2/M transition delay appears to be the mechanistic basis for the observed increased host cell invasion (Nguyen et al., 2016), since bacterial host invasion occurs mainly in the G2 phase (Alekseeva et al., 2013). Since all Lpl proteins share a highly conserved core sequence, there might be a common function that is accentuated by their multiplicity in a tandem gene cluster. Whether there is a correlation of G2/M phase transition delay and host cell invasion, and whether the one effect evokes the other, remains to be clarified by further studies. Further studies show that the Lpl of *S. aureus* can also induce in HeLa and osteoblast-like MG-63 cells breaks in DNA, mediated by alpha phenol-soluble modulins (PSMα1–4), as indicated by histone H2AX phosphorylation. In contrast, other Lpls encoded on pathogenicity island appear to depress H2AX phosphorylation to prevent DNA damage (Deplanche et al., 2019). Staphylococci isolated from the same patient over the course of disease (acute initial and recurrent bone and joint infections), were demonstrated to express lower amounts of Lpls resulting in more DNA-damage and G2/M transition delay suggesting involvement of these mechanisms in adaptive processes of bacteria during persistence (Deplanche et al., 2019). This broadens the understanding of mechanisms of *S. aureus*-host interaction and suggests its ability to cause persistent infections represents a balance between PSMα vs. LPLs expression (Deplanche et al., 2019). This insight into the mechanism of staphylococcal chronicity may provide a means of addressing the issue of treatment failure particularly in MRSA infected individuals.

Additional host cell invasion pathways of *S. aureus*, with direct involvement of Lpl1, have been identified using recombinant Lpl1 protein lacking the lipid moiety (Tribelli et al., 2020). This rhLpl1 binds directly to the human heat shock proteins HSP90α and HSP90ß isoforms. In addition**,** the Lpl1 peptide sequence potentiated *S. aureus* invasion of HaCaT cells two-fold to five-fold fold, while anti-HSP90 antibodies attenuated *S. aureus* invasion of HaCaT cells and primary human keratinocytes. In addition, the invasion of HaCaT cells was also depressed by inhibition of HSP90 ATPase functionality, and by siRNA silencing of HSP90α expression. Of the two isoforms, the inducible HSP90α appears to play a major role in Lpl1-host interaction, since its induction (increased temperature) correlated with both HSP90α expression and staphylococcal cell invasion. The essential host-staphylococcal interaction (Lpl1-HSP90) results in F-actin formation and resultant endocytosis.

Staphylococcal Lpls are also involved in the resistance of MRSA against almost all β-lactam antibiotics (Shang et al., 2019). Subinhibitory concentrations of β-lactam antibiotics induce upregulation of the cluster of lipoprotein-like genes, *lpl*, in MRSA. The increased expression of *lpl* by clinically used β-lactams is directly controlled by the global regulator *sarA,* a pleiotropic global regulator that modulates the expression of approximately 120 genes in *S. aureus* via *agr*-dependent or -independent pathways (Cheung et al., 2008). Of increased importance is that the β-lactam antibiotics induced elevated levels of *S. aureus* LPls and increased MRSA pathogenicity (Shang et al., 2019), a factor which may also contribute to reported treatment failure.

## Conclusion

The top leading cause of death worldwide in the last decades has remained heart disease, followed by cancer and COVID-19 (https://www.cdc.gov/nchs/fastats/leading-causes-of-death.htm; https://www.who.int/news-room/fact-sheets/detail/the-top-10-causes-of-death). Therefore, any effort for prevention and treatment of CVD and SARS-CoV-2 are important areas of research. HDL-Cholesterol (HDL-C) levels in plasma are inversely related to CVD risk, but most therapeutic approaches for increasing HDL-C in clinical trials have not shown benefit (Rader and Hovingh, 2014). It is postulated that it may be the function, rather than the cholesterol content of high-density lipoprotein (HDL) that accounts for its atheroprotection (Karathanasis et al., 2017).

HDLs are a complex mixture of particles with many different proteins and lipid components. Protective functions ascribed to HDL include reverse cholesterol transport from aortic foam cells to the liver, as well as anti-inflammatory, antioxidative, and antiprotease effects (Rye and Barter, 2014; Rosenson et al., 2016; Gordon and Remaley, 2017). Many of these functions of HDLs are performed by larger, spherical particles, so maturation of HDLs from small discoidal particles to large spherical particles may also be important for CVD protection (Asztalos et al., 2000; Asztalos et al., 2011; Karathanasis et al., 2017).

LCAT is an enzyme critical for HDL particle maturation (Ahsan et al., 2014). Currently, the therapeutic approaches/agents that have been or undergoing clinical trials are gene therapy, recombinant human LCAT (rhLCAT), and small molecules activators of LCAT (LCAT-targeted therapies (Yang et al., 2022). From the latter, the Daiichi-Sankyo compounds’ mechanism of activation of LCAT and their therapeutic activity with improvement of HDL functionality are, to date, the best characterized (Sasaki et al., 2021).

High levels of triacyclglycerols (TG) and triacylglycerol-rich lipoproteins (TGRLs) confer a residual risk of cardiovascular disease after optimal LDL-C–lowering therapy. Consensus is that LDL-C is a non-arguable primary target for lipid lowering treatment, but the optimization of TGRL for reducing the remnant risk of cardiovascular diseases is also an important aspect of CVD therapy. Potential targets for novel lipid lowering therapeutics have been identified, including the LDL receptor, PCSK9, the angiopoietin-like (ANGPTL) family, apolipoproteins (APOs) and LPL. These proteins have been the focus to develop novel therapeutics to treat hypertriacylglycerolemia in patients who do not reach the target goal of TG after using the currently available drugs, based on genetic studies of altered lipid phenotypes in the proteins regulating LPL activity, (Moon et al., 2022). A monoclonal antibody, Evinacumab, inhibiting ANGPTL3, is approved for use in the United States in patients with homozygous familial hypercholesterolemia. Volanesorsen inhibits APOC3 and was approved in Europe for the treatment of familial chylomicronemia syndrome, (Endocrinologic & Metabolic Drugs Advisory Committee, 2018. FDA Briefing Document:

EMDAC Meeting for Volanesorsen (Waylivra). Silver Spring: Food and Drug Administration; 2018. https://pink.pharmaintelligence.informa.com/-/media/supporting- documents/pink-sheet/2018/05/waylivrafda_back grounder.pdf?rev =b873388a6381495c9df19ee1e1895d3c&hash=E57C40 AEF44E4C8839C9542AA533EE5B; Gaudet et al., 2022). Other attempts to enhance LPL activity, by inhibiting ANGPTL3/8 complex with targeted monoclonal antibody therapy, showed dose dependent reduction in TG, LDL-C, non-HDL-C in phase 1 clinical trials (Gaudet et al., 2022). Beyond the abovementioned drugs, therapeutics have been attempted to lower LDL-C with different targets (Moon et al., 2022). Still, most of the studies appear to be focused on statin therapies which have a very narrow target population, e.g., those with homozygous familial hypercholesterolemia (Kim et al., 2022).

While all the attempts to develop agents directed to lowering the leading cause of human death in the world are necessary, it is essential to examine potential secondary effects activators of lipid enzymes associated with CVD, LCAT and LPL, have on host-pathogen interactions. There are many bacteria, fungi and protozoa that either highjack mammalian enzymes or produce LCAT- and LPL-like enzymes for survival and proliferation in the host, a factor which can complicate management of patients with co-morbidities. Monitoring patients in clinical settings, and patient education when any of the abovementioned therapies is prescribed, may be a crucial step in avoiding complications, e.g., chronic infection. In the last decade or so, drug repurposing saw an unprecedented utilization of drugs for off-targeted purposes. However, the development/use especially of covalent irreversible (human) enzyme modulators, must be taken with caution in situations where patients develop communicable diseases. The otherwise non-toxic to humans β-lactam antibiotics trigger the production of microbial lipase, LPL in MRSA. This in turn acts to promote increased virulence in *S. aureus.* The implication being that in situations where antibiotic resistance is reported, incidental use of lipid modulators for treatment of co-morbidities could potentially increase microbial virulence and therefore should be used with caution. Clearly more work needs to be performed in this area to better inform drug dosing and potential drug-lipid interactions.
